# Supplementary material for: Mechanisms governing the pioneering and redistribution capabilities of the non-classical pioneer PU.1
Source: Nat Commun. 2020 Jan 21;11:402. doi: 10.1038/s41467-019-13960-2 (PMC6972792; doi:10.1038/s41467-019-13960-2)
Supplement: Supplementary file 7 — Source data [file 41467_2019_13960_MOESM7_ESM.zip › Source_Data/Figure6/Figure6B_MotifScanOutput/ETS1_PU.1/homerResults/motif9.similar.html]

motif9

## Information for motif9

C
G
T
A
C
T
G
A
A
G
T
C
G
A
C
T
C
T
A
G
G
A
T
C
G
T
C
A
C
T
G
A
  
Reverse Opposite:  

G
A
C
T
C
A
G
T
C
T
A
G
A
G
T
C
C
T
G
A
A
C
T
G
A
G
C
T
A
C
G
T
  

|  |  |
| --- | --- |
| p-value: | 1e-211 |
| log p-value: | -4.877e+02 |
| Information Content per bp: | 1.709 |
| Number of Target Sequences with motif | 4430.0 |
| Percentage of Target Sequences with motif | 27.98% |
| Number of Background Sequences with motif | 5040.2 |
| Percentage of Background Sequences with motif | 15.72% |
| Average Position of motif in Targets | 99.0 +/- 55.8bp |
| Average Position of motif in Background | 99.4 +/- 66.4bp |
| Strand Bias (log2 ratio + to - strand density) | -0.0 |
| Multiplicity (# of sites on avg that occur together) | 1.12 |
| Motif File: | file (matrix) reverse opposite |

### Similar de novo motifs found

|  |  |  |  |  |  |  |  |
| --- | --- | --- | --- | --- | --- | --- | --- |
| Rank | Match Score | Redundant Motif | P-value | log P-value | % of Targets | % of Background | Motif file |
| 1 | 0.876 | C A T G C T G A A G T C C T A G A C T G A C G T A C G T | 1e-106 | -246.161014 | 37.92% | 27.93% | motif file (matrix) |
| 2 | 0.921 | G A C T A C T G C T A G G T A C C T G A C T A G G A C T C G A T T C A G T C G A | 1e-92 | -213.428653 | 19.82% | 12.59% | motif file (matrix) |
